# Experimental and Computational Evidence of a Stable RNA G‐Triplex Structure at Physiological Temperature in the SARS‐CoV‐2 Genome

**DOI:** 10.1002/anie.202415448

**Published:** 2024-11-06

**Authors:** Marco Campanile, Roberto Improta, Luciana Esposito, Chiara Platella, Rosario Oliva, Pompea Del Vecchio, Roland Winter, Luigi Petraccone

**Affiliations:** ^1^ Department of Chemical Sciences University of Naples Federico II Via Cintia 4 80126 Naples Italy; ^2^ Institute of Biostructure and Bioimaging National Research Council CNR Via P. Castellino 111 80131 Naples Italy; ^3^ Physical Chemistry I—Biophysical Chemistry Department of Chemistry and Chemical Biology TU Dortmund University Otto-Hahn Street 4a 44227 Dortmund Germany

**Keywords:** Biophysics, G-quadruplex, G-triplex, Molecular Modelling, SARS-CoV-2

## Abstract

RG1 is a quadruplex‐forming sequence in the SARS‐CoV‐2 genome proposed as possible therapeutic target for COVID‐19. We demonstrate that the dominant conformation of RG1 under physiological conditions differs from the parallel quadruplex previously assumed. Through comprehensive investigations employing CD, UV, NMR, DSC, gel electrophoresis, MD simulations, in silico spectroscopy and the use of truncated RG1 sequences, we have identified this stable conformation as an RNA G‐triplex composed of two G‐triads. We believe this previously unreported RNA structure could serve as a novel therapeutic target. Our findings open new avenues for further studies on the presence and biological role of RNA G‐triplexes in vivo.

## Graphical abstract

The quadruplex‐forming RG1 sequence in the SARS‐CoV‐2 genome is shown to adopt a two‐triads G‐triplex structure at physiological temperature. The high thermodynamic stability of this RNA structure suggests that RNA G‐triplexes could be biologically relevant and represent novel targets for antiviral therapies.

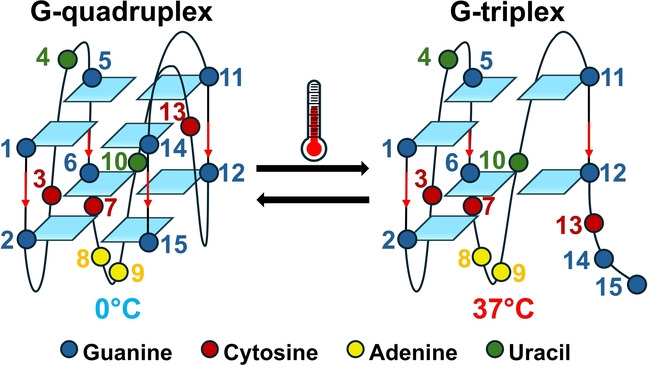

DNA and RNA G‐rich sequences can form noncanonical G‐quadruplex (G4) structures, formed by stacking of G‐tetrad layers of four guanines locked by Hoogsteen‐type hydrogen bonds and further stabilized by monovalent cations such as K^+^ or Na^+^.^[1]^ The formation of such structures is involved in the regulation of gene expression as well as in many other cellular processes, including RNA metabolism, epigenetic states, and related diseases.[^2^, ^3^, ^4^] Moreover, the biological role of G4s is not limited to humans but extends to yeasts, bacteria, and viruses.^[5]^ Therefore, G4s emerged as promising therapeutic targets for drug development.[^3^, ^5^, ^6^, ^7^] Bioinformatics analysis has been widely used to search for potential G‐quadruplex forming sequences in the genomes of several organisms.[^8^, ^9^, ^10^, ^11^] The minimal requirement for a G‐rich sequence to be a putative G‐quadruplex forming sequence is to contain four G‐tracts (regions of at least two adjacent guanines). However, a G‐rich sequence containing only three G‐tracts can potentially form a G‐triplex, another non‐canonical structure strictly related to the G‐quadruplex.[^12^, ^13^] G‐triplexes are formed by several layers of G‐triads each containing Hoogsteen‐like hydrogen bonds similar to the ones forming a G‐tetrad.[^12^, ^13^, ^14^, ^15^] Although G‐triplexes share similarities with G‐quadruplexes, they offer unique structural and functional properties that deserve further investigation. Putative G‐triplex sequences clearly include the number of the putative G‐quadruplex sequences in a genome but reasonably exceed this number. Understanding their role in biological processes and their potential as therapeutic targets could open new avenues for drug development. Despite their potential biological role, studies on G‐triplexes are extremely limited. The formation of a stable G‐triplex structure has been demonstrated for few DNA sequences[^13^, ^14^, ^15^, ^16^, ^17^] and is often invoked as intermediate in DNA G‐quadruplex folding/unfolding processes.[^18^, ^19^, ^20^] To our knowledge, stable G‐triplexes have never been reported for an RNA sequence.

In this work, we investigated in detail the conformational behavior of the RG1 sequence (GGCUGGCAAUGGCGG), an RNA G‐rich sequence in the SARS‐CoV‐2 genome located in the coding sequence region of the viral nucleocapsid phosphoprotein (denoted N‐protein).^[21]^ Previous experimental and computational studies suggested that RG1 forms a monomolecular two G‐tetrads parallel quadruplex.[^21^, ^22^] Remarkably, our data reveals that RG1 forms a stable RNA G‐triplex structure at physiological temperature whereas the corresponding G‐quadruplex conformation is not significantly populated.

We firstly characterized the RG1 conformation in the presence of 10 mM phosphate buffer pH 7.4 and 100 mM of K^+^ (K^+^ buffer) at 0 °C, conditions that should stabilize a folded G‐quadruplex conformation. The thermal UV/Vis difference spectrum (TDS) profile of RG1 is indeed characteristic of a G4 structure with maxima at 248 nm and 274 nm and a minimum around 297 nm (Figure 1A). Further, the value of the TDS factor ΔA240 nm/ΔA295 nm >4 is consistent with the parallel topology.^[23]^ The circular dichroism (CD) spectrum is also consistent with a parallel G‐quadruplex with a maximum at 268 nm and a minimum at 239 nm (Figure 1B, black spectrum).^[21]^ We further checked the molecularity of RG1 by means of native polyacrylamide gel electrophoresis (PAGE) experiments using as references the two G‐tetrads TBA and the three G‐tetrads Tel‐24 monomolecular quadruplexes.^[24]^ The gel electrophoresis results clearly show that RG1 migrates as a single band up to 100 μM strand concentration with a mobility similar to TBA (Figure 1C). Overall, our UV, CD and PAGE results indicate that RG1 forms a monomolecular two‐G‐tetrads parallel G‐quadruplex in agreement with a previously proposed structural model.^[22]^

**Figure 1:**
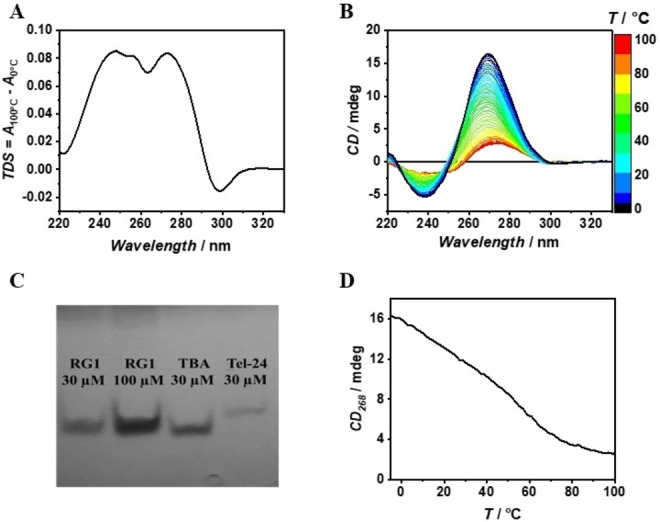
TDS (A), 3D melting (B), Native PAGE (C) and CD melting curve (D) of RG1. All the samples were prepared in K^+^ buffer.

To verify whether this structure is stable at physiological temperature, we then recorded the 3D melting profile by means of temperature‐dependent CD measurements (Figure 1B). Remarkably, the CD melting profile at 268 nm shows a multiphasic shape, suggesting that RG1 unfolding is not a simple two‐state process (Figure 1D). To determine the number of significant species needed to properly analyze the thermal unfolding, all the collected temperature dependent spectra were subjected to Singular Value Decomposition (SVD) procedure.^[25]^ SVD analysis clearly reveals that at least three main conformations are involved in the whole RG1 unfolding process (see section 8.1 in the Supporting Information for further discussion). We further verified the complexity of the RG1 unfolding by following the process by means of Differential Scanning Calorimetry (DSC). As expected, the obtained DSC profile clearly shows two transitions centered at about 25 °C and 60 °C confirming the biphasic behavior of the RG1 unfolding (Figure 2C). On the basis of DSC, CD and SVD results, we modeled the RG1 unfolding with a three‐states mechanism (Figure 2A) involving an intermediate species (see section 8.2 in the Supporting Information for details). The proposed mechanism matched the experimental melting profiles with great accuracy (Figure 2B,C). Inspection of the thermodynamic parameters (Table 1) reveals that RG1 unfolds with a total enthalpy change of 210 kJ/mol, a value in agreement with the one expected for a two G‐tetrads RNA quadruplex.[^26^, ^27^] Notably, about 65 % of the enthalpy and entropy change is involved in the unfolding of the intermediate state suggesting that it is a well‐structured species somehow resembling the RG1 G‐quadruplex. Figure 2D shows the molar fraction of each species as function of temperature. Interestingly, the intermediate species represents by far the most populated one at physiological temperature (>75 %). This is significant as this RG1 conformation could be the most suitable target for a future antiviral strategy. For this reason, we explored the nature of the intermediate species in the RG1 unfolding in more detail.

**Figure 2:**
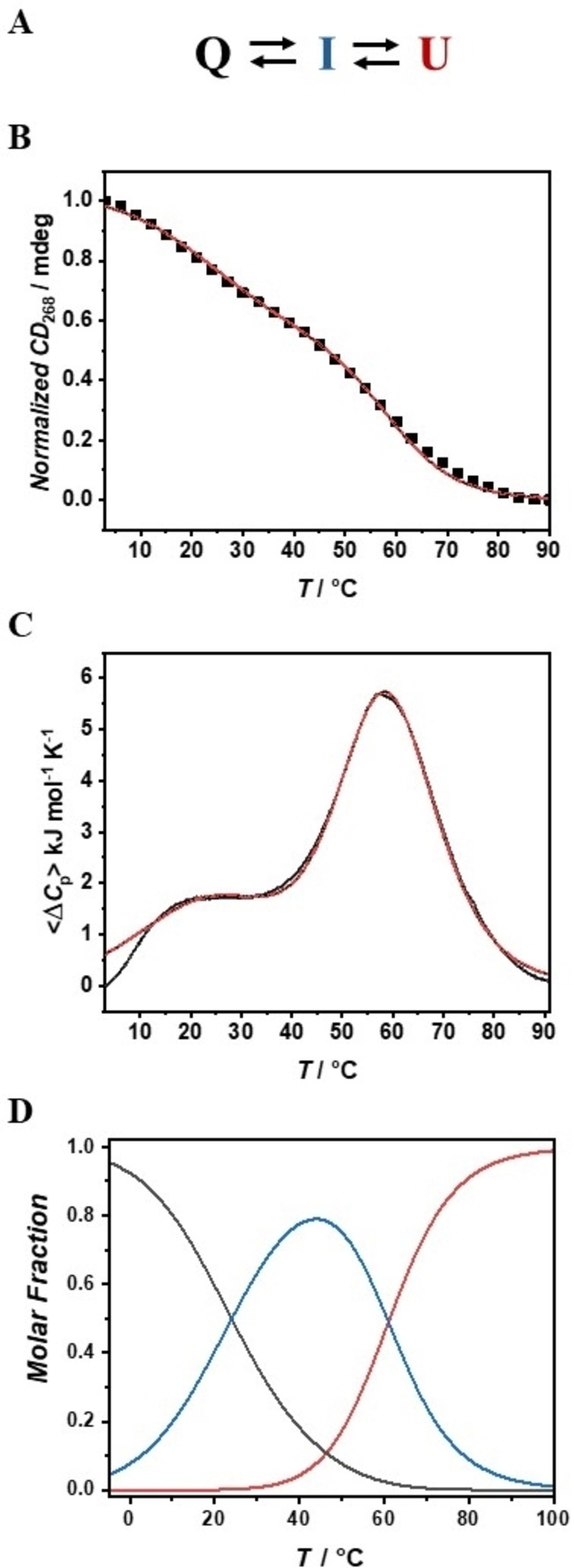
Three‐states mechanism for the RG1 unfolding (A). CD (B) and DSC melting experiments (C); red lines represent the best fitting curves (with adjusted R^2^ >0.99). Temperature‐dependent molar fractions (D).

**Table 1:** Thermodynamic parameters for the RG1 unfolding obtained from the global fit analysis of the CD and DSC data

*Transition*	T/°C	ΔH°/kJ mol ^1^	ΔS°/kJ K^−1^ mol^−1^
*Q→I*	26±2	71±8	0.24±0.03
*I→U*	59.2±0.4	139±12	0.42±0.04
*Q→U*	\	210±14	0.66±0.05

First, we recorded the TDS spectrum, setting as a lower limit the temperature at which the intermediate state (**I**) of RG1 is at the highest molar fraction (∼40 °C), so that the resulting TDS should mainly reflect the structural features of this state. Interestingly, the TDS does not show the minimum at 297 nm (present at 0 °C) revealing that **I** is not a quadruplex structure (Figure 3A). We then determined the CD spectrum of **I** by knowing the experimental CD spectra of the quadruplex and unfolded RG1 and the molar fraction of each species as a function of temperature (see section 8.3 in the Supporting Information for details). The spectrum of **I** (Figure 3B) is remarkably similar to that of the RG1 quadruplex revealing that the intermediate species has a structure similar to the one of the parallel G‐quadruplex. However, its CD intensity is about 40 % lower than the one of the RG1 quadruplex, suggesting that the intermediate state is not composed of fully formed parallel G‐tetrads. All these observations argue in favor of a G‐triplex as a reasonable structural candidate for **I**, as such structure could be virtually obtained by removing just two consecutive guanine bases from the G‐quadruplex. The resulting parallel G‐triplex, as observed in our experiment, should have a similar shape but a less intense CD spectrum.^[17]^ To support our hypothesis, we simulated by means of an hybrid QM/MM approach (see section 10.1 in the Supporting Information for computational details) the expected CD spectral changes for a G‐quadruplex—G‐triplex transition assuming that the CD spectrum of each species is mainly due to the G‐core. Briefly, we simulated the ECD spectrum for the two model systems depicted in Figure 3C, containing two guanine tetrads (2×G4‐K^+^) or two triads (2×G3‐K^+^) coordinating one central K^+^ ion. As Figure 3B shows, the simulated spectra well reproduce the differences between the spectra of RG1 quadruplex and **I**. Remarkably, the computed spectra of 2×G4‐K^+^ and 2×G3‐K^+^ have a similar shape but the intensity of the former is ∼45 % higher, in agreement with the observed intensity change experimentally observed. We also verified that the introduction of the deoxyribose backbone and the other bases do not qualitatively change the electronic circular dichroism (ECD) spectra (see section 10.2 in the Supporting Information for further discussion). To prove the existence of G‐triads in the species **I**, we looked for the presence of Hoogsteen hydrogen bonds by recording ^1^H NMR spectra over the 15–40 °C temperature range (Figure 3D and Figure S3). The observed signals in the 10.4–11.5 ppm range are consistent with the ones expected for imino protons characteristic of the presence of G‐tetrads and/or G‐triads.[^12^, ^14^] Interestingly, the imino signals at 40 °C are more resolved than at 15 °C, thus suggesting a reduction of the conformational heterogeneity of RG1 on increasing the temperature, in agreement with the calculated molar fractions (Figure 2D). Notably, the persistence of signals in the 10.4–11.5 ppm range and the appearance of a new signal at 10.4 ppm on increasing the temperature are fully consistent with a G‐quadruplex—G‐triplex transition with the latter becoming the prevalent conformation at 40 °C.[^14^, ^19^] To gain additional insights into the guanines involved in the G‐triplex formation, we characterized the truncated sequences of RG1, 3‐RG1 and 5‐RG1, obtained by removing two consecutive guanines from the 3’ or 5’ end respectively. Both truncated sequences form, under our experimental conditions, a monomolecular structure with an electrophoretic mobility comparable to that of RG1 (see Figure S4).

**Figure 3:**
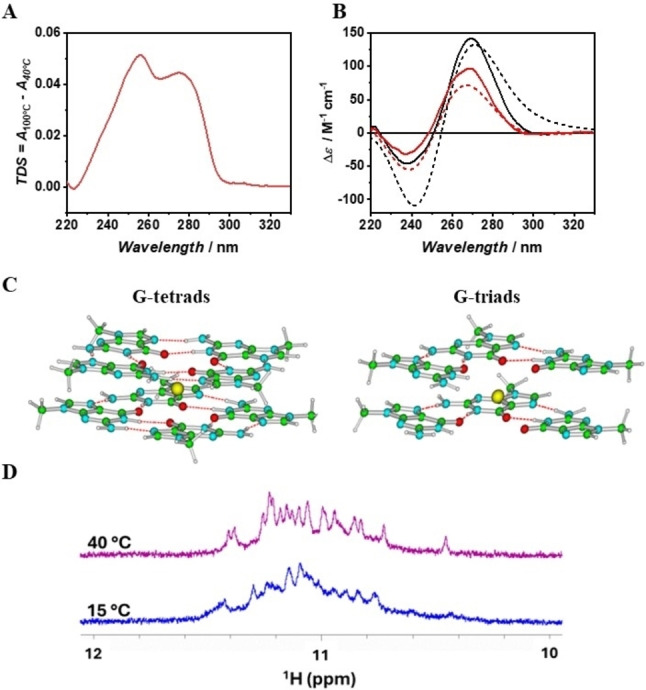
TDS of the intermediate species (A). Experimental (continuous lines) and theoretical (short dash lines) CD spectra of the RG1 quadruplex (in black) and its unfolding intermediate (in red) (B). Schematic drawing of the G4X2‐K^+^ (on the left) and G3X2‐K^+^ (on the right) models (C). ^1^H NMR spectra of RG1 in the region of Hoogsteen imino peaks (D).

To note that these truncations cannot form an intramolecular G‐quadruplex but could potentially form an intramolecular G‐triplex. Remarkably, the spectrum of 3‐RG1 is almost superimposable to the spectrum of the intermediate species of RG1 unfolding, whereas the 5‐RG1 spectrum has a lower intensity and an additional negative peak at about 292 nm (Figure 4A). Moreover, the 3‐RG1 shows a cooperative two‐state unfolding with increasing temperature whereas 5‐RG1 does not, suggesting that 3‐RG1 forms a well folded compact structure (Figure 4B). Further, the 3‐RG1 unfolding enthalpy is remarkably close to the enthalpy changes for the RG1 intermediate species unfolding (Figure S5). Overall, these results suggest that the main conformation of the 3‐RG1 sequence is very similar to the one of the intermediate state in the RG1 unfolding. Note that 3‐RG1 is a very short oligonucleotide (13 nucleotides long) and it is difficult to predict intramolecular structures other than a G‐triplex capable of accounting for its CD spectrum and the observed unfolding enthalpy changes. The conformational behavior of both 3‐RG1 and 5‐RG1 was further explored by means of molecular dynamics (MD). The MD simulations confirm that 5‐RG1 is significantly less structured than 3‐RG1, which adopts a compact structure where some Hoogsteen base pairings are maintained between adjacent strands, including a significant population (>40 %) of G‐triplex (Figure 4C, see also section 10.3 in the Supporting Information for further details). Further, the predicted ECD spectra for the most populated cluster extracted from the MD simulations are indeed consistent with the experimental CD spectrum of 3‐RG1 (Figure S10 and S11). Overall, both our experimental and computational results reveal that the intermediate species in RG1 unfolding is a G‐triplex formed by the detachment of the two 3’‐end guanines from the corresponding G‐tetrads.

**Figure 4:**
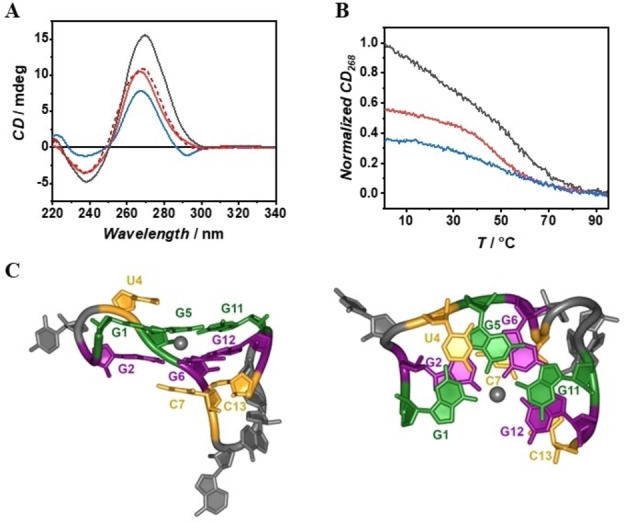
CD spectra of the RG1 quadruplex (black curve), 3‐RG1 (red curve), experimental RG1 intermediate (red dashed curve) and 5‐RG1 (blue curve) (A). Normalized CD melting curves of RG1 (black curve), 3‐RG1 (red curve) and 5‐RG1 (blue curve) (B). Orthogonal views of 3‐RG1 G‐triplex structure obtained by MD analysis. The K+ ion is depicted as a grey sphere. For clarity, only sugar and base rings are shown as sticks (C).

Noteworthy, the RG1 G‐triplex is much more stable than the DNA two‐triads G‐triplex previously reported^[14]^ whose melting temperature is below the physiological temperature, suggesting that RNA G‐triplexes may be intrinsically more stable and biological relevant than the DNA G‐triplex. Further, the observed capability of RG1 to adopt two alternative non‐canonical nucleic acids structures can be the basis of a more complex regulatory switch as previously proposed for DNA.^[28]^ In summary, our data unambiguously demonstrate that under physiological conditions the dominant conformation of RG1 is different from the parallel quadruplex previously assumed but it forms an RNA G‐triplex containing two‐triads. Such previously unreported RNA structure could represent a new antiviral target. Since G‐rich regions are highly abundant in human mRNAs, telomeric RNA and in viral RNA genomes, our study paves the way for further investigations into the presence of RNA G‐triplex in vivo and their biological role.

## Supporting Information

The authors have cited additional references within the Supporting Information.[^29^, ^30^, ^31^, ^32^, ^33^, ^34^, ^35^, ^36^, ^37^, ^38^, ^39^, ^40^, ^41^, ^42^, ^43^, ^44^, ^45^, ^46^, ^47^, ^48^, ^49^, ^50^, ^51^, ^52^, ^53^, ^54^, ^55^, ^56^]

## Conflict of Interests

The authors declare no conflict of interest.

## Supporting information

As a service to our authors and readers, this journal provides supporting information supplied by the authors. Such materials are peer reviewed and may be re‐organized for online delivery, but are not copy‐edited or typeset. Technical support issues arising from supporting information (other than missing files) should be addressed to the authors.

Supporting Information

## Data Availability

The data that support the findings of this study are available in the supplementary material of this article.
