# Supplementary material for: Experimental and Computational Evidence of a Stable RNA G‐Triplex Structure at Physiological Temperature in the SARS‐CoV‐2 Genome
Source: Angew Chem Int Ed Engl. 2024 Nov 6;63(52):e202415448. doi: 10.1002/anie.202415448 (PMC11656141; doi:10.1002/anie.202415448)
Supplement: Supplementary file 1 — Supporting Information [file ANIE-63-e202415448-s001.pdf]

Angewandte Chemie
Eine Zeitschrift der Gesellschaft Deutscher Chemiker

Supporting Information

Experimental and Computational Evidence of a Stable RNA G-Triplex Structure at Physiological Temperature in the SARS-CoV-2 Genome

*M. Campanile, R. Improta, L. Esposito, C. Platella, R. Oliva, P. Del Vecchio, R. Winter, L. Petraccone**

Evidence of a stable RNA G-triplex structure at physiological temperature in the SARS-CoV-2 genome

Marco Campanile[1], Roberto Improta[2], Luciana Esposito[2], Chiara Platella[1], Rosario Oliva[1], Pompea Del Vecchio[1], Roland Winter[3] and Luigi Petraccone[1]*

[1]Department of Chemical Sciences, University of Naples Federico II, Via Cintia 4, 80126 Naples, Italy.
[2]Institute of Biostructure and Bioimaging, CNR, Via P. Castellino 111, 80131 Naples, Italy.
[3]Physical Chemistry I – Biophysical Chemistry, Department of Chemistry and Chemical Biology, TU Dortmund University, Otto-Hahn Street 4a, Dortmund 44227, Germany.

*To whom correspondence should be addressed. Tel:+39081674263 Email:luigi.petraccone@unina.it

Supplementary Material

Summary

Table of content ... 3
1. Materials ... 4
2. Samples preparation ... 4
3. UV-Vis spectroscopy ... 4
4. Circular Dichroism (CD) ... 4
5. Native Polyacrylamide Gel Electrophoresis (PAGE) ... 4
6. Differential Scanning Calorimetry (DSC) ... 5
7. Nuclear Magnetic Resonance (NMR) ... 5
8. Data Analysis ... 5
8.1 Singular Value Decomposition (SVD) ... 5
8.2 Q ⇄ I ⇄U model and global fit ... 8
8.3 Calculation of the intermediate CD spectrum from experimental data ... 9
9. Additional figures for the experimental part ... 10
10. QM and MD ... 12
10.1 Computational details ... 12
10.1.1 QM/MM calculations ... 12
10.1.2 Simulation of the ECD spectra ... 12
10.1.3 MD simulations. ... 12
10.2 Results of QM and QM/MM calculations ... 14
10.2.1 RG1 ... 14
10.2.2 RG1 truncated sequence at the 3'end (3-RG1) ... 16
10.3 MD simulations ... 17
10.3.1 RG1 ... 17
10.3.2 RG1 G-triplex structures obtained by deleting two terminal Gs ... 19
References ... 24

Table of content

Figure S1. Plot of the first 10 singular values obtained through the SVD analysis as function of the spectral component…………………………………………………………………………………………..6

Table S1. Singular values, values for the first-order autocorrelation of the U and V matrices and relative variances obtained for the first 10 spectral components. ……………………………………………………7

Figure S2. Data matrix **D** obtained from the 3D melting of the RG1 and residual matrices (**RES**) calculated by considering only 2 or 3 significant components…………………………………………………………..7

Figure S3. Imino proton regions of the ^{1}H NMR spectra of RG1…………………………………………..10

Figure S4. Native PAGE of RG1 truncations in the presence of 10 or 100 mM KCl………………………..10

Figure S5. CD melting of the 3-RG1 3.4 µM in K^+ buffer and results of vant'Hoff analysis………………...11

Figure S6. Schemes of the RG1 quadruplex and triplexes…………………………………………………13

Figure S7. Schematic drawing of the computational model used in QM/MM ONIOM calculations on RG1, based on the results of MD simulations…………………………………………………………………14

Figure S8. Computed ECD spectra forG4X2-K^+ at the PCM/TD-M052X/6-31G(d) level and from RG1 at PCM/QM/MM level, using either AMBER or UFF force fields for the MM part……………………………..15

Figure S9. Schematic drawing of the computational model used in QM/MM ONIOM calculations on the structural model from 3-RG1, based on the results of MD simulations………………………………………..16

Figure S10. Computed ECD spectra for RG1 and the most populated structure for the 3-RG1 according to MD simulations……………………………………………………………………………………………17

Figure S11. Computed ECD spectra for RG1 and the most populated structure for the 3-RG1 sequence according to MD simulations. Only the Gua bases are considered in the calculations, keeping the same arrangement provided by MD simulations…………………………………………………………………17

Figure S12. MD simulation of RG1 at room temperature. (A) Time evolution of RMSD from the starting structure and schematic representation of the representative RG1-GQ structure……………………………...18

Figure S13. Schematic representation of tetrad and triad planes with Hoogsteen H-bonds…………………..19

Table S2. Percentage of persistence of Hoogsteen tetrad H-bonds along the 1 µs trajectory…………………19

Figure S14. Time evolution of RMSD from the starting structure of 3-RG1 and 5-RG1……………………...20

Figure S15. Time evolution of the total number of Hoogsteen H-bonds along the 13-mer trajectories………..21

Table S3. Fraction of Hoogsteen H-bonds along the 150 ns trajectories of the 13-mer models………………22

Figure S16. Time evolution of gyration radius (Rgyr) in 3-RG1 and 5-RG1 simulations……………………22

1. Materials

The RNA sequences RG1 (GGCUGGCAAUGGCGG), 3-RG1 (GGCUGGCAAUGGC), 5-RG1 (CUGGCAAUGGCGG) and the DNA sequence TBA (GGTTGGTGTGGTTGG) were provided by *GenScript Biotech* (Piscataway, NJ, USA) as HPLC grade lyophiles. The DNA sequence Tel-24 (TTAGGGTTAGGGTTAGGGTTAGGG) was purchased by *Eurogentec* (Seraing, Belgium). RNAse free water, berberine hydrochloride, EDTA disodium salt dihydrate, potassium chloride (KCl), potassium dihydrogen phosphate (KH_2PO_4), potassium monohydrogen phosphate (K_2HPO_4), Acrylamide/bis-acrylamide (29:1) 40% solution, glycerol, GelRed Nucleic Acid Stain, Ammonium persulfate (APS) and Tetramethylethylenediamine (TEMED) were purchased by *Merck* (Darmstadt, Germany).

2. Sample preparation

RNA stock solutions were prepared by dissolving the powder in RNAse free water. The concentration was spectrophotometrically determined by recording the absorbance at 260 nm and using the nearest-neighbor model derived extinction coefficients of 143800, 123100, 123500 M^{-1} cm^{-1} for the RG1, 3-RG1 and 5-RG1, respectively. The samples were then diluted at the desired concentration in the presence of 10 mM potassium phosphate (KP) buffer pH 7.4, 100 mM KCl and 0.2 mM EDTA (K^+ buffer), heated at 95 °C for 4 minutes and left to slowly cool to room temperature prior to storage at 4 °C and use.

3. UV-Vis spectroscopy

Temperature-dependent spectra of the RG1 were collected on a Jasco V770 equipped with a Peltier for temperature control from *Jasco Corporation* (Tokyo, Japan). The sequence at a concentration of 4.2 µM was previously annealed in the K^+ buffer and loaded in a 1 cm path-length quartz-cuvette. All the spectra were recorded between 220 and 330 nm with a scan rate of 100 nm/min and 2 accumulations were performed on each spectrum. Firstly, the sequence was equilibrated at 20 °C for 10 minutes, then it was slowly cooled to 0 °C for the recording of the reference spectrum for the quadruplex conformation. The next spectra were collected after heating at 40 °C and 100 °C. Thermal differences spectra (TDS) were calculated by subtracting the spectrum at 0 °C or 40 °C from the spectrum at 100 °C.

4. Circular Dichroism (CD)

CD experiments were performed on a Jasco J1500 spectropolarimeter equipped with a Peltier for temperature control from *Jasco Corporation* (Tokyo, Japan). All the spectra were recorded in a 1 cm quartz cuvette between 220 and 340 nm, with a scan rate of 50 nm/min, a D.I.T. of 2 s and a bandwidth of 4 nm. 3D melting experiments on the RG1 sequence were conducted by increasing the temperature with a ramp of 1 °C/min in the range 0 to 100 °C and recording a spectrum every 2 °C. 2D melting curves were recorded from 0 to 100 °C at 1 °C/min by monitoring the signal at 268 nm.

5. Native Polyacrylamide Gel Electrophoresis (PAGE)

To prepare a 20% polyacrylamide gel, a 40% solution of acrylamide:bis-acrylamide 29:1 was diluted in TBE 1x as running buffer. The polymerization was conducted between two 20 cm x 20 cm slabs

with a spacing of 0.5 cm. The electrophoresis experiments were run at 20 °C for 4 h with an applied constant voltage of 120 V. For the sample loading, 30 µL of previously annealed samples in K^+ buffer were mixed with 20 µL of a loading buffer composed of TBE 10x and 20% v/v glycerol. Gel visualization was conducted by means of UV-shadowing when the oligonucleotides concentrations employed were > 30 µM. To determine the RG1 molecularity, we compared its electrophoretic mobility to a monomolecular G-quadruplex of similar weight (TBA) and an almost as twice heavier G-quadruplex (Tel-24). For the experiments performed at lower concentrations the gel was stained for 1 h with the fluorescent dye GelRed diluted in the running buffer according to the manufacturer instructions. Visualization was performed with a UV-transilluminator ChemiDoc XRS from *Bio-Rad* (Hercules, CA, USA).

6. Differential Scanning Calorimetry (DSC)

Calorimetric experiments for the study of the RG1 unfolding were performed on a NanoDSC III from *TA Instruments* (New Castle, DE, USA). The total oligonucleotide concentration was 300 µM in K^+ buffer. The temperature interval was 0 to 100 °C, the scanning speed was set at 1 °C/min and the pressure at 3 atm. At least three heating and cooling scans were performed to evaluate the reversibility of the unfolding process. A buffer vs. buffer scan was subtracted from the measurements prior to the analysis with the NanoAnalyze software provided by the manufacturer.

7. Nuclear Magnetic Resonance (NMR)

NMR data were collected on Bruker Ascend 400 MHz NMR spectrometer from *Bruker Corporation* (Billerica, MA, USA) in the range 15-40 °C. Oligonucleotide samples were prepared in 10 mM KP buffer, 100 mM KCl, 90%/10% H_2O/D_2O, at 0.6 mM oligonucleotide concentration per strand. NMR spectra were acquired with the use of the DPFGSE solvent suppression method. DSS (4,4-dimethyl-4-silapentane-1-sulfonic acid) was used as a reference to calibrate the chemical shifts. NMR spectra were processed and analysed with the use of TopSpin 4.3.0 (Bruker).

8. Data Analysis

Graph plots and data analysis were performed on Origin 2018 from *OriginLab Corporation* (Northampton, MA, USA) and/or MATLAB R2021b from *MathWorks* (Natick, MA, USA).

8.1 Singular Value Decomposition (SVD)

To identify the number of significant spectral components involved in the RG1 thermal unfolding, an SVD analysis was performed as previously described.[1] SVD is a method from linear algebra for factoring a matrix.[2] Briefly, the matrix of the CD spectra D is decomposed by the SVD method into the product of three matrices:

$$\boldsymbol{D = USV^t}$$

The matrix U contains the basis spectra which combine to form the experimentally observed CD spectra, S is a diagonal matrix that contains as many singular values as the number of basis spectra in the U matrix. V is a matrix containing the coefficient vectors as function of temperature. The magnitude of each singular value represents the contribution (weight) of the corresponding basis spectrum to the observed spectral behavior (contained in the data matrix). The singular values start relatively high and then decrease to a low noisy plateau which represents the noise level. The magnitudes of the singular values provide the first clue of the number of significant basis spectra.

Another criterion used for determining the number of significant spectral components was the value of the first-order autocorrelation function for the columns of the U and V matrices that should be close to 1 to indicate nonrandom behavior (i.e. “signal”). An additional indication of the number of significant spectral species was provided by the evaluation of the contribution of each singular value to the total variance in singular values. The relative variance (RV) of each singular value is given by:

$$\boldsymbol{RV} = \frac{\sum_i \boldsymbol{S}_i^2}{\sum_i \boldsymbol{S}_i^2}$$

where S_i^2 is the square of the singular value. Only the significant singular values will sum to contribute to >0.99 of the total variance. We found that the first 3-4 singular values appear to significantly deviate from the remaining S values (Figure S1). This observation, together with the analysis of the magnitudes of the autocorrelation factors of the V and U matrices and of the relative variance (RV) associated with each S value (Table S1) reveals that at least three main conformations are involved in the whole RG1 unfolding processes. To further confirm this result, we computed the difference between the primary CD melting data and the reproduced CD spectra obtained by using the first two or three basis spectra obtained from SVD analysis (Figure S2):

$$\boldsymbol{RES} = \boldsymbol{D} - \boldsymbol{U}\boldsymbol{S}_{\boldsymbol{red}}\boldsymbol{V}^{\boldsymbol{t}}$$

where $\mathbf{S}_{\mathbf{red}}$ includes the first two or three singular values (all the other diagonal elements of S are set to 0). Inspection of the residuals plots clearly shows that merely two spectral components are not enough to describe the whole spectral behavior (as the residual significantly deviates from the random noise) and that a third spectral component is needed to have a residual close to the noise level (Figure S2, panel C)

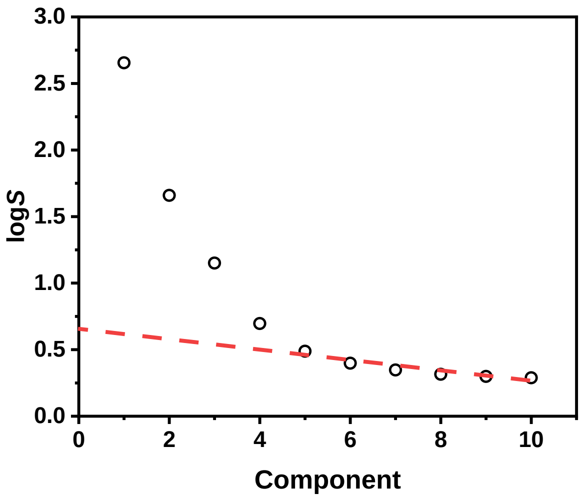

Figure S1. Plot of the first 10 singular values obtained through the SVD analysis as a function of the spectral component. The dashed red line is a guide for the eye to highlight the deviation of the S values from the smooth decrease representing the noise level.

Table S1. Singular values, values for the first-order autocorrelation of the U and V matrices and relative variances obtained for the first 10 spectral components.

i	$S_{i,i}$	U autocorrelation	V autocorrelation	Relative Variance
1	452.404695	0.999123319	0.974168981	0.988481249
2	45.61247294	0.998178715	0.967771643	0.010048043
3	14.15381944	0.991950152	0.8900209	0.000967524
4	4.972115241	0.925230413	0.824808226	0.000119398
5	3.077969523	0.929895431	-0.103607747	4.58E-05
6	2.499497704	0.874125639	-0.252982696	3.02E-05
7	2.222942658	0.812719608	0.068567204	2.39E-05
8	2.067353302	0.731656549	-0.016964888	2.06E-05
9	1.989558699	0.782016383	-0.239577748	1.91E-05
10	1.943852975	0.813176838	-0.016170676	1.82E-05

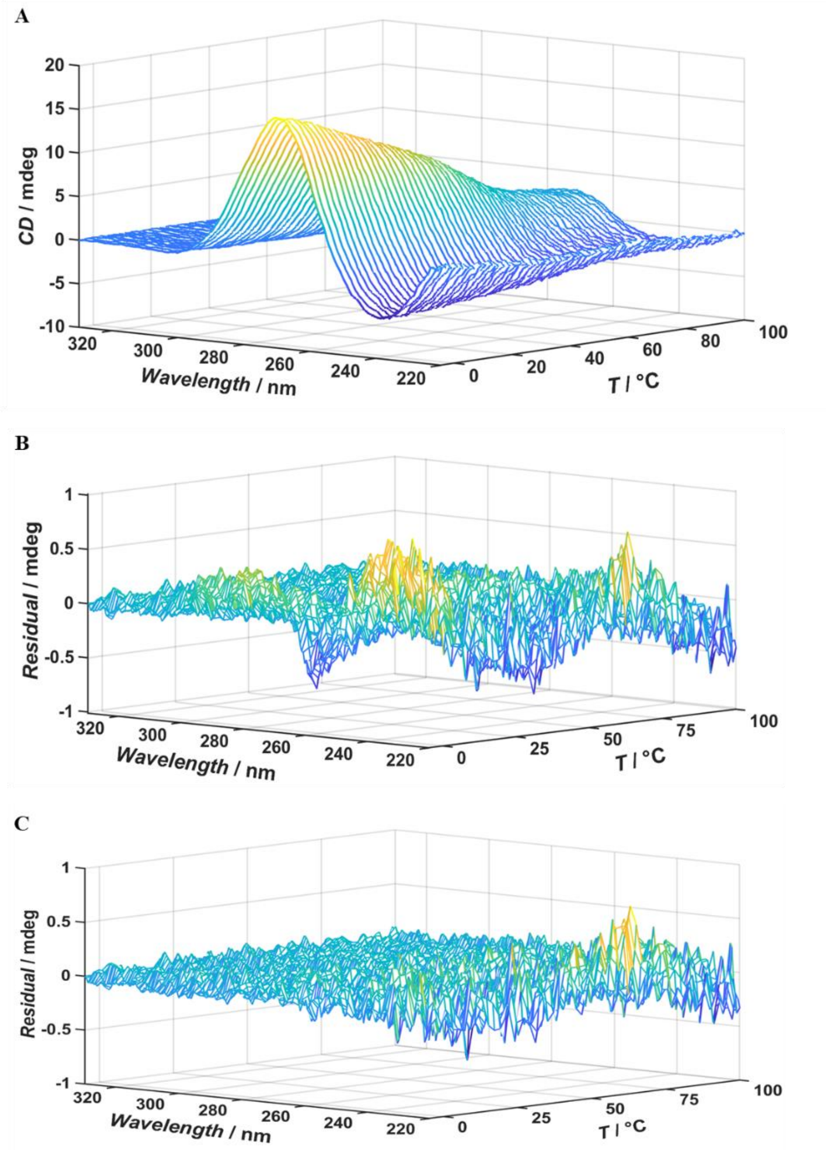

Figure S2. Data matrix **D** obtained from the 3D melting of the RG1 (A). Residual matrices (**RES**) calculated by considering only 2 (B) or 3 (C) significant components.

8.2 Q ⇄ I ⇄U model and global fit

The complex thermal unfolding of the RG1 sequence was then modelled according to the following scheme of equilibria:

$$Q \rightleftarrows I \rightleftarrows U$$

where Q, I and U are the quadruplex, intermediate and unfolded species, respectively. To obtain the thermodynamic parameters from the experimental CD melting and DSC thermogram, two independent functions were derived from the three-states model:

1. 2D CD melting:

$$CD_{normalized}(T) = CD_Q \cdot \frac{1}{1 + k_1(T) + k_1(T)k_2(T)} + CD_I \cdot \frac{k_1}{1 + k_1(T) + k_1(T)k_2(T)}$$

with:

$$k_1(T) = e^{-\frac{\Delta H^\circ_{Q\to I}(\frac{1}{T}-\frac{1}{T_1})}{R}}$$

$$k_2(T) = e^{-\frac{\Delta H^\circ_{I\to U}(\frac{1}{T}-\frac{1}{T_2})}{R}}$$

where CD(T) is the CD signal at a given temperature normalized between 1 and 0, CD_Q and CD_I are the normalized CD values corresponding to the quadruplex and intermediate conformation, respectively; the normalized CD signal for the unfolded sequence, CD_U, is assumed 0; T_1 and $\Delta H^\circ_{Q\to I}$ and T_2 and $\Delta H^\circ_{I\to U}$ are the transition temperatures and enthalpy changes for the Q ⇄ I and the I ⇄ U equilibrium, respectively.

2. DSC thermogram:

$$< \Delta C_p > (T) = \frac{\Delta H^{\circ 2}_{Q\to I} \cdot F_I + \Delta H^{\circ 2}_{Q\to U} F_U - (\Delta H^\circ_{Q\to I} \cdot F_I + \Delta H^\circ_{Q\to U} F_U)^2}{RT^2}$$

with:

$$F_I = \frac{k_1}{1 + k_1(T) + k_1(T)k_2(T)}$$

$$F_U = \frac{k_1 k_2}{1 + k_1(T) + k_1(T)k_2(T)}$$

where $< \Delta C_p >$ is the excess constant-pressure heat capacity at a given temperature and $\Delta H^\circ_{Q\to U} = \Delta H^\circ_{Q\to I} + \Delta H^\circ_{I\to U}$ is the total enthalpy change for the RG1 unfolding. The heat capacity changes ΔC_p° for the conversion among the different conformations are assumed to be negligible. The other parameters have the same meaning as in the CD equation.

The equations derived from the three-states model were implemented in the software Origin 2018 to perform a global fit of the CD and DSC data. The transition temperatures T_1 and T_2, and the enthalpy changes $\Delta H^\circ_{Q\to I}$ and $\Delta H^\circ_{I\to U}$ were set as shared and adjustable parameters. In addition, only for the CD

expression, both the CD_Q and the CD_I were set as adjustable with $CD_Q \geq 1$. All the thermodynamic quantities which better describe the RG1 unfolding were then determined by fitting the experimental data. The entropic terms $\Delta S^{\circ}_{Q\rightarrow I}$ and $\Delta S^{\circ}_{I\rightarrow U}$ were calculated as $\frac{\Delta H^{\circ}_{Q\rightarrow I}}{T_1}$ and $\frac{\Delta H^{\circ}_{I\rightarrow U}}{T_2}$, respectively.

8.3 Calculation of the intermediate CD spectrum from experimental data

The thermodynamic quantities obtained from the global fit were employed to calculate the molar fractions of the species Q, I and U at every temperature in the range -10 to 110 °C. The CD spectrum at each temperature is given by the equation:

$$CD(T) = CD_Q * f_Q(T) + CD_I * f_I(T) + CD_U * f_U(T)$$

where CD_Q, CD_I and CD_U are the CD spectra corresponding to 100% quadruplex, intermediate or unfolded RG1 structure. Hence CD_I can be calculated by rearranging the equation as:

$$CD_I = \frac{CD(T) - CD_Q * f_Q(T) - CD_U * f_U(T)}{f_I(T)}$$

The equations were implemented in a MATLAB script to compute the intermediate spectrum at each experimental temperature and using each CD spectrum recorded in the 3D melting. The references for the quadruplex and unfolded conformations were set as follows: $CD_Q = CD_{(0°C)}$ and $CD_U = CD_{(100°C)}$. Only the temperatures for which the molar fraction of the intermediate was ≥ 0.10 were taken into consideration. Consequently, a matrix containing an intermediate spectrum for each temperature considered was obtained. The final spectrum CD_I was obtained by averaging over all the spectra.

9. Additional figures for the experimental part

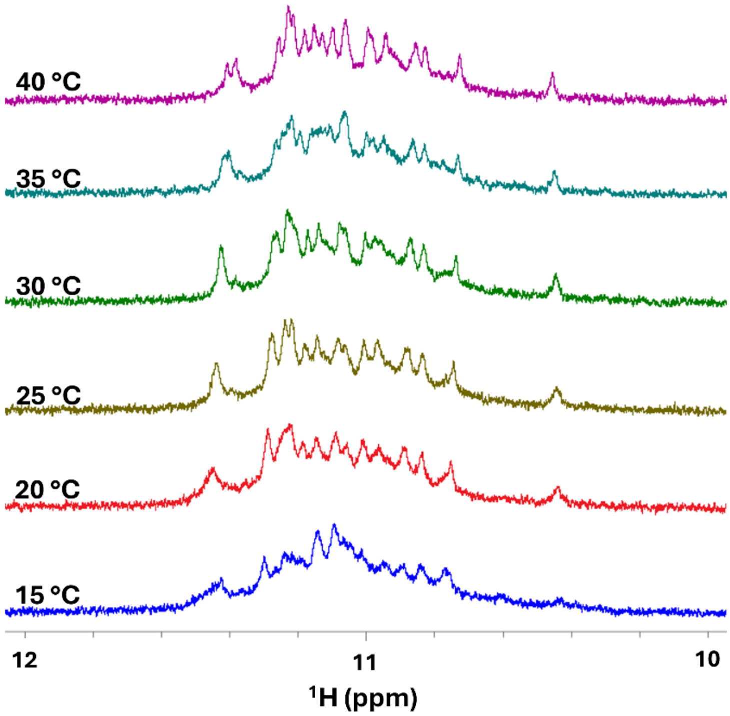

Figure S3. Imino proton regions of the ¹H NMR spectra of RG1. The spectra were recorded at 0.6 mM oligonucleotide concentration in 10 mM KP buffer, 100 mM KCl, 90%/10% H_2O/D_2O, in the range 15-40 °C. Temperatures are shown on the left of the corresponding spectrum.

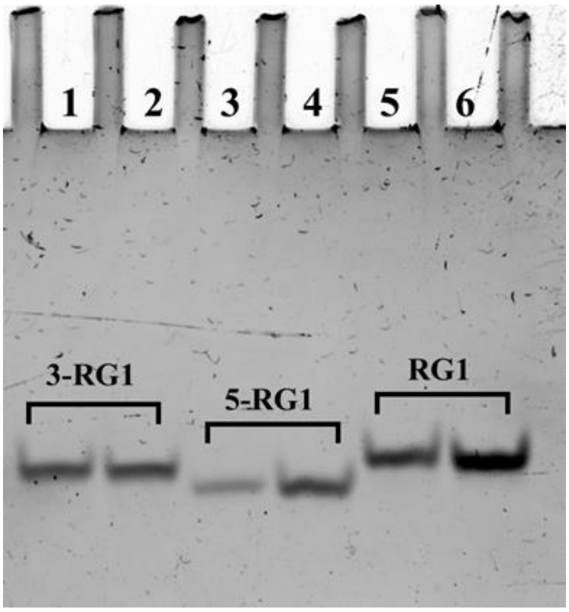

Figure S4. Native PAGE of the indicated sequences in the presence (lanes 1, 3, 5) or absence (lanes 2,4,6) of 100 mM KCl. The gel was stained with the GelRed dye and visualized with a UV-transilluminator.

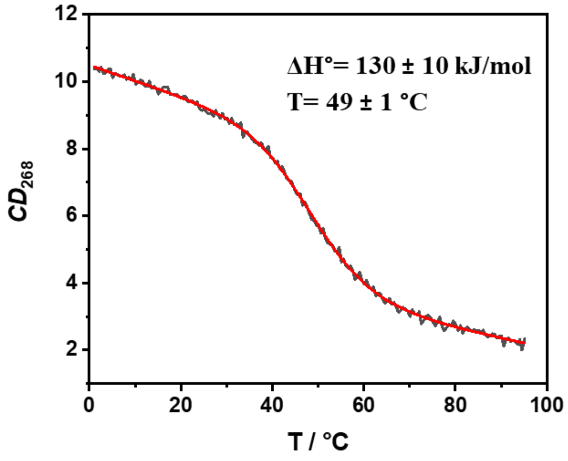

Figure S5. CD melting of the 3-RG1 3.4 μM in K^+ buffer (black curve) and line of best fit obtained through a vant'Hoff analysis assuming a simple two-states unfolding model (red curve).

10. QM and MD

10.1 Computational details

10.1.1 QM/MM calculations

The ground electronic state of the different computational models was optimized using Quantum Mechanics/Molecular Mechanics (QM/MM) calculations. Density Functional Theory (DFT) was used as QM method, selecting the M052X functional and using the 6-31G(d) basis set. M052X functional, thanks to its accurate description of dispersion interactions and charge transfer electronic transitions,[3] has been already profitably used to study the photophysics and the photochemistry of closely stacked polynucleotides,[4,5] including guanine quadruplexes (GQ).[6] In particular, it well reproduces the lineshape of the electronic circular dichroism spectra of guanine quadruplex of different topologies, except for an almost uniform blue-shift of the spectra (see below).[7–9]

The MM part was described using the Amber force field as implemented in the Gaussian16 program,[10] parm21.dat with the OL3 update.[11] We also performed test calculations with UFF force fields obtaining very similar spectra. QM and MM calculations were coupled by using the ONIOM implemented in the Gaussian16 program.

The solvent effect was included by using the Polarizable Continuum Model (PCM),[12] using CPCM model in QM/MM geometry optimization[13] and the IEFPCM model in the calculation of the spectra.[14]

10.1.2 Simulation of the ECD spectra

The ECD spectra were simulated by computing the 60 lowest energy excited states at the PCM/QM/MM level, using for the QM part Time-Dependent DFT, with the M052X functional and the 6-31G(d) basis set. Then each stick contribution to the spectrum was broadened with a phenomenological gaussian with half-width half-maximum 0.33 eV. As anticipated above, the resulting ECD spectrum is significantly blue-shifted with respect to the experimental one, confirming previous indications.[7–9] Actually, when this procedure is applied to simulate the absorption spectrum of 9methyl-guanine, the maximum of the resulting absorption spectrum is blue shifted by 0.80~0.85 eV with respect to the experimental absorption spectrum of dGMP in water.[15] Such shift is partially (0.1~0.2 eV) due to the absence in our treatment of vibrational effects,[16] while the main part is related to all the source of inaccuracies in our computational approach (density functional, basis set, solvation model etc). In previous studies, we have shown that, the description of the monomer, and not the treatment of the electronic interactions in the GQ, is the main source of error in the spectra computed by using our procedure.[7,17] As a consequence, in order to allow an easier comparison with the experimental spectrum, the simulated spectrum was shifted by -0.85 eV. This is the same shift used in our previous computational studies of CD spectra of GQ.[9]

10.1.3 MD simulations.

We simulated the RG1 sequence in explicit water, with the Amber22 package,[18] the OL3 RNA force field,[11,19,20] the TIP3P water model,[21] and the Joung and Cheatham ion parameters.[22] We started from the model of a parallel GQ structure proposed by Miclot et al. (GR1-rep.c0.pdb extracted from the paper Supplementary Material) which consists in a two-quartet GQ structure with one potassium ion approximately at the centre of the two tetrad planes (Figure S6A).[23] We ran a 1 μs simulation at

300 K to verify the structural stability of the quadruplex arrangement. We also investigated two reduced RG1 sequences obtained by deleting the two terminal Gs at either the 3'end (3-RG1) or the 5'end (5-RG1) (Figure S6B,C). The starting structure was derived from the same model of the whole sequence with the two Gs omitted. We ran five independent 500 ns simulations for each sequence at 300 K. Since the 13-mer RG1 oligonucleotides (3-RG1 and 5-RG1) were less stable than the whole 15-mer sequence, the analysis was limited to the first 150 ns of each simulation.

All the models were solvated in a truncated octahedral box with a minimal distance of 10 Å of solute from the box border. Potassium counterions were used to neutralize the charge of the systems and KCl was added to reach a concentration value of 150 mM in order to reproduce usual experimental conditions. Periodic boundary conditions were imposed in all three dimensions and the time step was set to 2 fs. The starting structures were energy minimized using both steepest descent and conjugated gradient algorithms (500 + 500 steps). Energy minimization was followed by the heating step (80 ps) from 100 to 300 K carried out by restraining the GQ molecule by a harmonic potential. Equilibration of each system was first conducted for 200 ps at a 300 K temperature (NVT ensemble) by gradually releasing the positional restraints on the GQ molecule. Unrestrained systems were then simulated for 100 ps in an NPT ensemble at 1 atm of pressure using the Langevin equilibration scheme to keep the temperature constant. After equilibration, production MD runs for RG1 sequences were performed at 300 K in the NPT ensemble with the Monte Carlo barostat used for the pressure control.[24] The particle mesh Ewald (PME) was used to compute the electrostatic interactions.[25] For Lennard-Jones interactions, a cut-off of 9 Å was applied. Bond lengths involving hydrogen atoms were constrained using the SHAKE algorithm.[26] An integration time step of 2 fs was used and structures were recorded every 10 ps collecting a total of 100000 snapshots along the simulation time.

Analyses of trajectories were performed using the cpptraj module of AMBER,[27] and the VMD (version 1.9.3) program which was used for visualization.[28] PyMol (version 1.8.2.0) and Grace (version 5.1.22) programs were used to produce graphs and pictures.[29]

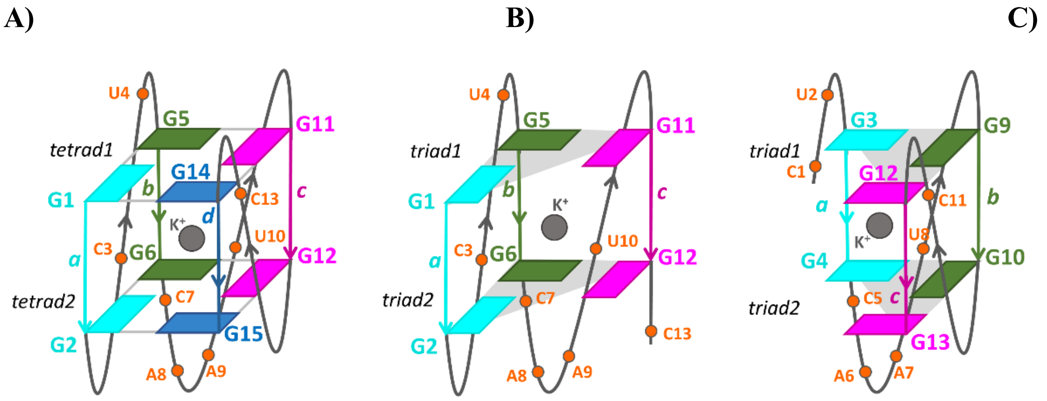

Figure S6. Scheme of the RG1 quadruplex and triplexes. Starting from the 5'-end, the four parallel strands of G nucleotides are denoted as *a* (cyan), *b* (green), *c* (purple), and *d* (blu). (A) Two-tetrads RG1-GQ; (B) Two-triads 3-RG1 model; (C) Two-triads 5-RG1 model. The nucleotides in the loops are highlighted as orange circles.

10.2 Results of QM and QM/MM calculations

10.2.1 RG1

As discussed in the main text, according to both experiments at low temperature and MD simulations the 8 guanine bases of RG1 are arranged in a parallel quadruplex (GQ) composed by two tetrads (G4X2) coordinating one central K^+ ion (G4X2-K^+). At the same time, there are several experimental indications that the intermediate of RG1 predominant at physiological temperature exhibits a triplex-like arrangement (GT) with six G bases coordinating one K^+ ion (G3X2-K^+). Considering that the number of studies analysing the consequences of a GQ→GT transitions on the ECD spectrum is still limited, as a first step of our computational analysis we have thus computed the ECD spectrum of ideal GQ and GT systems in a parallel topology. To this aim we have optimized the models depicted in Figure 3C of the main text, including only guanine bases, arranged as in parallel GQ, and where the sugar is replaced by a methyl group. The G4X2 model has been already profitably used in the first QM study analysing the spectral properties of GQ.[30] These models were then fully optimized in water at the PCM/M052X/6-31G(d) level and their CD spectra computed at the PCM/TD-M052X/6-31G(d) level (see Figure 3B in the main text). As discussed in the main text, the ECD spectrum of G4X2-K^+ is typical of a parallel GQ, and the differences between the spectra of G4X2-K^+ and G3X2-K^+ are fully consistent with those existing between RG1 and Int1 intermediate.

As a next step, we considered the effect of the deoxyribose backbone and of the other bases, by performing ONIOM QM/MM geometry optimizations using the structures issuing from MD simulations as starting point. Our computational model is depicted in Figure S7, the Guanine bases are treated at the QM level (also including the methyl group bonded at N9 atom), while the other atoms at the MM level. In the QM/MM minimum the G bases keep a parallel arrangement and the resulting spectrum is very similar to that obtained on the smaller G4X2 model, as shown in Figure S8, and to the experimental one of RG1. Moreover, the spectra computed by using AMBER or UFF force fields are very similar, indicating that our conclusions are solid with respect to this choice.

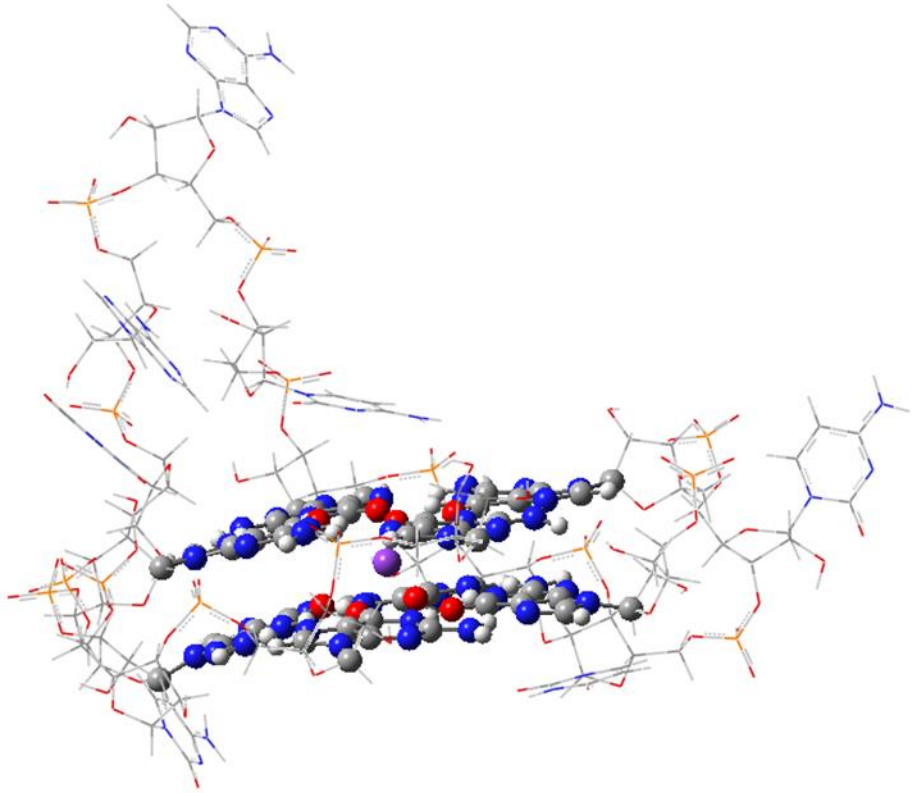

Figure S7. Schematic drawing of the computational model used in QM/MM ONIOM calculations on RG1, based on the results of MD simulations. The bases treated at the QM level are represented by ball and sticks, those treated at the MM level only by lines. Colour code: Blue: Nitrogen atoms; Red oxygen atoms; Grey: Carbon atoms; White: Hydrogen atoms; Yellow: Phosphor atoms; Purple: K^+ ion.

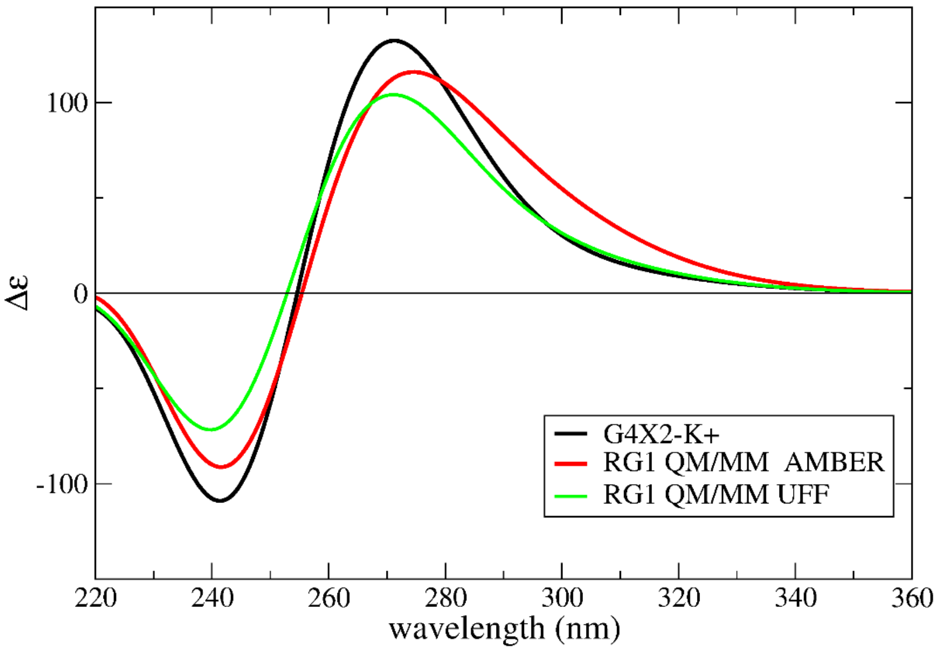

Figure S8. Computed ECD spectra forG4X2-K^+ at the PCM/TD-M052X/6-31G(d) level (black spectra) and from RG1 at PCM/QM/MM level, using either AMBER (red spectrum) or UFF (green spectrum) force fields for the MM part. Δε in 10^{-40} esu^2 cm^2. Spectra obtained by shifting the contribution of each excited state by -0.85 eV and convoluting it with a Gaussian with half width half maximum = 0.33 eV.

In our computational model solvent molecules are not explicitly considered, as well as the counterions, with the exception of the K^+ ion coordinated by the two tetrads. Moreover, some bases are treated also at the QM level, whereas other only at the MM level, and this unbalance can lead to some spurious results (i.e. the geometry optimization can show the tendency to maximize the interactions between the bases treated at the QM level). Finally, being based on a single minimum, conformational effects are not considered. Shortly, basing our conclusions only on the outcome of QM/MM geometry optimizations could not be safe. In parallel, considering that the main scope of our computational analysis is to assist the interpretation of the experimental ECD spectra, we thus resorted also to another procedure. Starting from the average structures issuing from MD simulations, we extracted the guanine bases and the coordinated K^+ ion, and we optimized their inner geometrical parameters (bond angles and bond length), in order to avoid possible artefacts related to the use of MM geometries, but freezing the values of the dihedral angles, in order to maintain the same average arrangement predicted by MD simulations. In other words, we computed the spectrum of a 4GX2-K^+ system where the mutual orientation of the base is the same as in the RNA sequence. The results obtained by this approach are denoted by the suffix -gua. In Figure S11 below we show the spectrum computed for RG1-gua, which is very similar to that predicted for RG1, but less intense.

10.2.2 RG1 truncated sequence at the 3'end (3-RG1)

Experiments and MD simulations indicate that 5-RG1 is not well structured, whereas the experimental features of 3-RG1 are similar to those of the intermediate predominant at physiological temperature. In order to get some insights in this issue we studied the most representative cluster extracted from the MD simulations on 3-RG1 (Figure S9) and discussed in detail in section 10.3 section the analysis of the MD simulations.

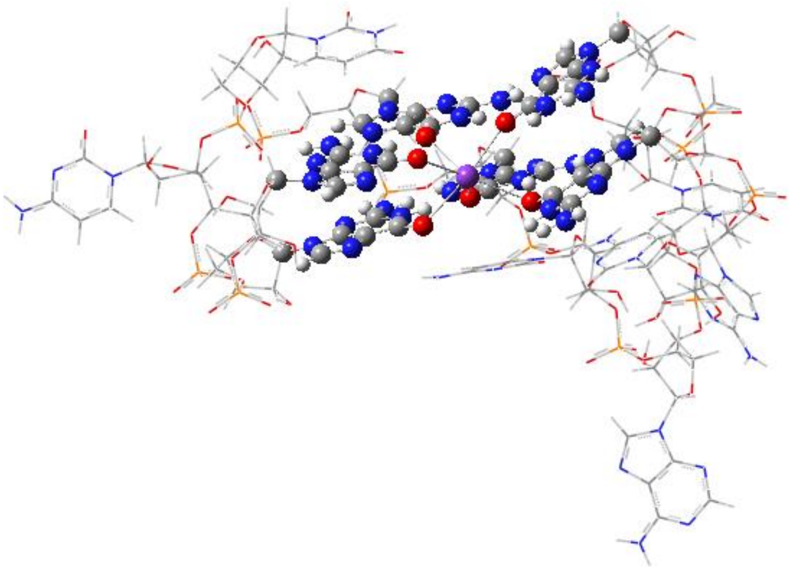

Figure S9. Schematic drawing of the computational model used in QM/MM ONIOM calculations of the structural model from 3-RG1, based on the results of MD simulations. The bases treated at the QM level are represented by ball and sticks, those treated at the MM level only by lines. Colour code: Blue: Nitrogen atoms; Red oxygen atoms; Grey: Carbon atoms; White: Hydrogen atoms; Yellow: Phosphor atoms; Purple: K^+ ion.

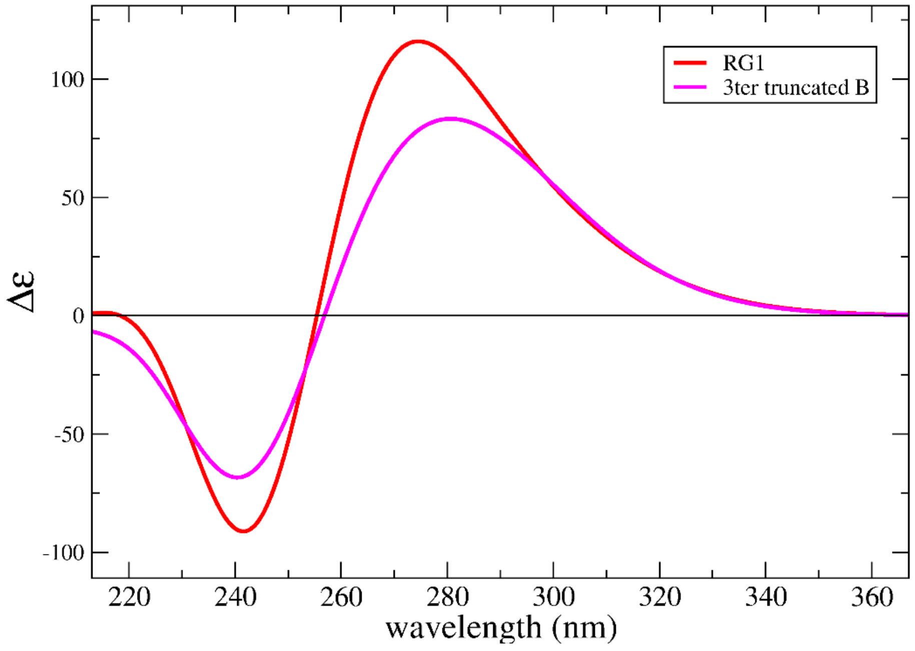

Figure S10. Computed ECD spectra for RG1 (red) and the most populated structure for the 3-RG1 according to the MD simulations (magenta). PCM/TD-M052X/631G(d)/AMBER ONIOM calculations. Δε in 10^{-40} esu^2 cm^2. Spectra obtained by shifting the contribution of each excited state by -0.85 eV and convoluting it with a Gaussian with half width half maximum = 0.33 eV.

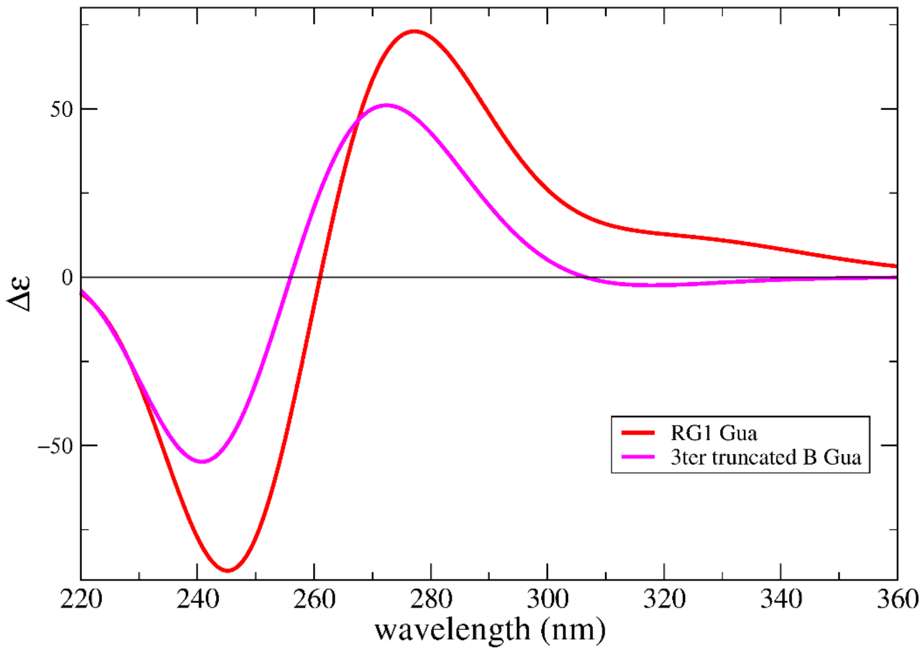

Figure S11. Computed ECD spectra for RG1 (red) and the most populated structure for the 3-RG1 sequence according to the MD simulations (magenta). Only the Gua bases are considered in the calculations, keeping the same arrangement provided by the MD simulations. PCM/TD-M052X/631G(d) calculations. Δε in 10^{-40} esu^2 cm^2. Spectra obtained by shifting the contribution of each excited state by -0.85 eV and convoluting it with a Gaussian with half width half maximum = 0.33 eV.

The ECD spectra computed for RG1 and the most representative structure of its 3'ter truncated sequence (3-RG1) are reported in Figures S10 (all the systems were studied at the QM/MM level) and S11 (only the Gua bases are considered). The MD derived 3-RG1 structure has similar shape to the experimental ECD spectrum of RG1, with a smaller intensity of the positive peak, as found for the 3-RG1 species in the experimental spectra. Moreover, especially when only the bases are considered (but keeping the same arrangement issuing from MD simulations, see Figure S11) a small blue-shift of the maximum is obtained, further improving the agreement with experiments. This analysis thus supports our proposal that the 3-RG1 adopts a G-triplex-like conformation in solution.

10.3 MD simulations

10.3.1 RG1

The GQ parallel structure consisting of two quartets (Tetrad1 formed by G1, G5, G11, G14; Tetrad2 formed by G2, G6, G12, G15) is very stable along the whole 300 K trajectory showing a RMSD around 1 Å calculated on the eight G nucleotides that form the tetrads (Figure S12A). Indeed, by considering the hydrogen bonds connecting the G bases in each tetrad (Figures S13A and S6A), on average the persistence of them along the trajectory is equal to 96% and 93% for tetrad1 and tetrad2, respectively (Table S2). A representative structure for RG1 GQ was calculated by averaging on all

atoms along the trajectory and then extracting the structure closest to average which is represented in Figure S12B.

A)

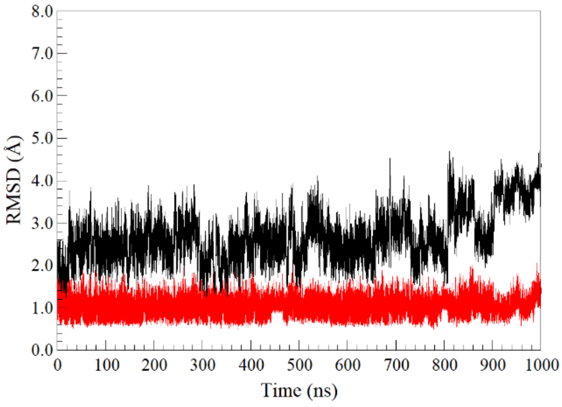

B)

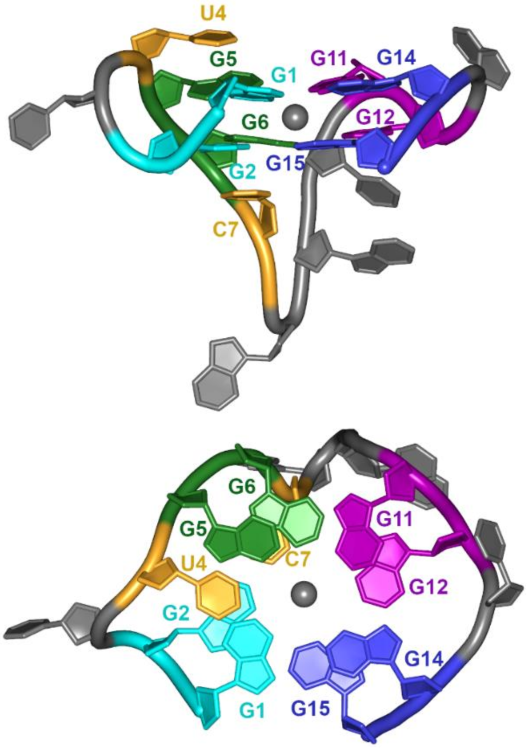

Figure S12. MD simulation of RG1 at room temperature. (A) Time evolution of RMSD from the starting structure. The RMSD is calculated for all nucleotide atoms of the 15mer model (black), and for G nucleotides only(red); (B) Schematic representation of the representative RG1-GQ structure (close to the average) in two orthogonal views. The Gs in the four parallel strands are coloured differently in strand a (cyan), b (green), c (purple), and d (blue); the stacked bases in the loop regions are highlighted in bright orange. The K^+ ion is depicted as a grey sphere. For clarity, only sugar and base rings are shown as sticks.

A)

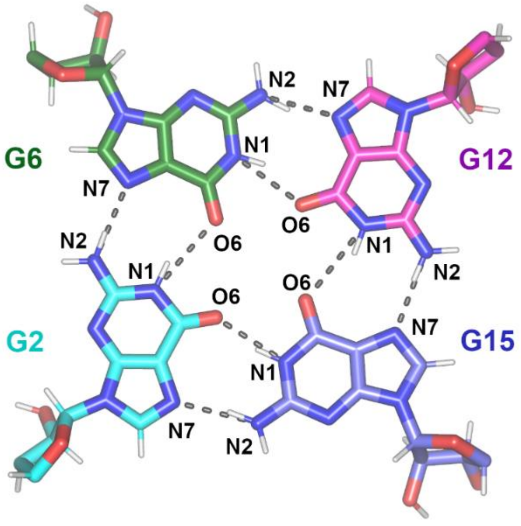

B)

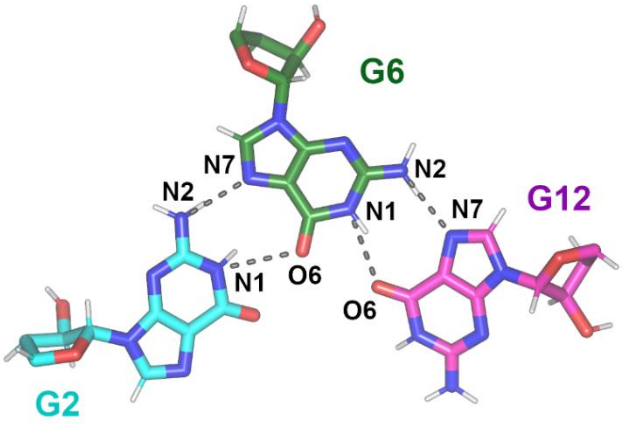

Figure S13. Schematic representation of tetrad and triad planes with Hoogsteen hbonds. (A) Example of tetrad (tetrad2) from RG1 representative structure (close to the average calculated on the T = 300 K trajectory); (B) Example of triad (triad2) from 3-RG1 representative structure. Only bases and sugars are shown in sticks representation.

Table S2. Percentage of persistence of Hoogsteen tetrad H-bonds along the 1 μs trajectory.

**H-bond***		**% Persistence**
Tetrad1		
**G1-G5**	N1...O6	95.5
**G1-G5**	N2...N7	97.2
**G5-G11**	N1...O6	93.4
**G5-G11**	N2...N7	94.0
**G11-G14**	N1...O6	96.9
**G11-G14**	N2...N7	96.1
**G14-G1**	N1...O6	94.4
**G14-G1**	N2...N7	97.0
Tetrad2		
**G2-G6**	N1...O6	88.2
**G2-G6**	N2...N7	99.0
**G6-G12**	N1...O6	89.4
**G6-G12**	N2...N7	92.2
**G12-G15**	N1...O6	87.7
**G12-G15**	N2...N7	96.5
**G15-G2**	N1...O6	92.4
**G15-G2**	N2...N7	97.8

* The H-bond routine to calculate the occurrence of the interactions was applied by using the following cutoffs for donor (D)-acceptor (A) distance and angle: d_{AD} < 3.3 Å, angle DH...A > 135°.

10.3.2 RG1 G-triplex structures obtained by deleting two terminal Gs

From RG1-GQ we built two new 13mer models by deleting either the two 5'end Gs (5-RG1) or the two 3'end Gs (3-RG1), thus obtaining structures formed by two G-triads (planar units formed by three H-bonded guanosines). We performed five independent simulations for each model (Figure S6B,C) at 300K for a length of 150 ns. Under the same conditions used for the GQ simulation none of the models remained stable for the entire simulation (Figure S14).

A) **B)**

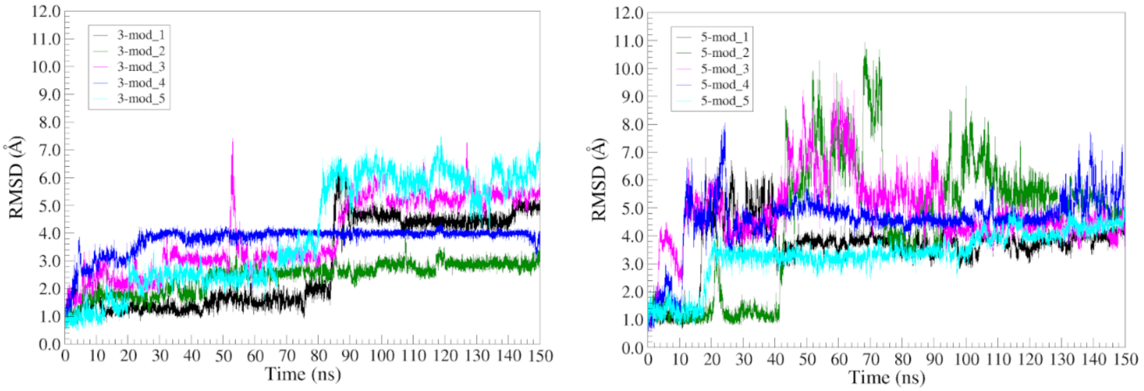

Figure S14. Time evolution of RMSD from the starting structure. The RMSD is calculated for all G nucleotide atoms of the two-triad 13mer models for the five independent MD runs. (A) 3-RG1 simulations; (B) 5-RG1 simulations.

The longest living G-triplex structure was found in the 3-RG1_1 simulation where it lasts about 80 ns. Although marked perturbations of the triads are observed for both 3-RG1 and 5-RG1 models, a difference can be found between the two systems. Indeed, by analysing the persistence of the eight hydrogen bonds (four for each triad plane) along the trajectories it results that they are more conserved for the 3-RG1 simulations (see Table S3, Figures S13B, S15). This indicates that even when the G-triplex structure is not fully formed, the system adopts a compact structure where some base pairings are maintained between adjacent strands. In particular, the most stable pairings occur between the strand containing the 5'end (strand a) and its adjacent strand (strand b) (Table S3). The higher compactness of the 3-RG1 compared to the 5-RG1 structures is also evident from the time evolution of the gyration radius along the trajectories (Figure S16).

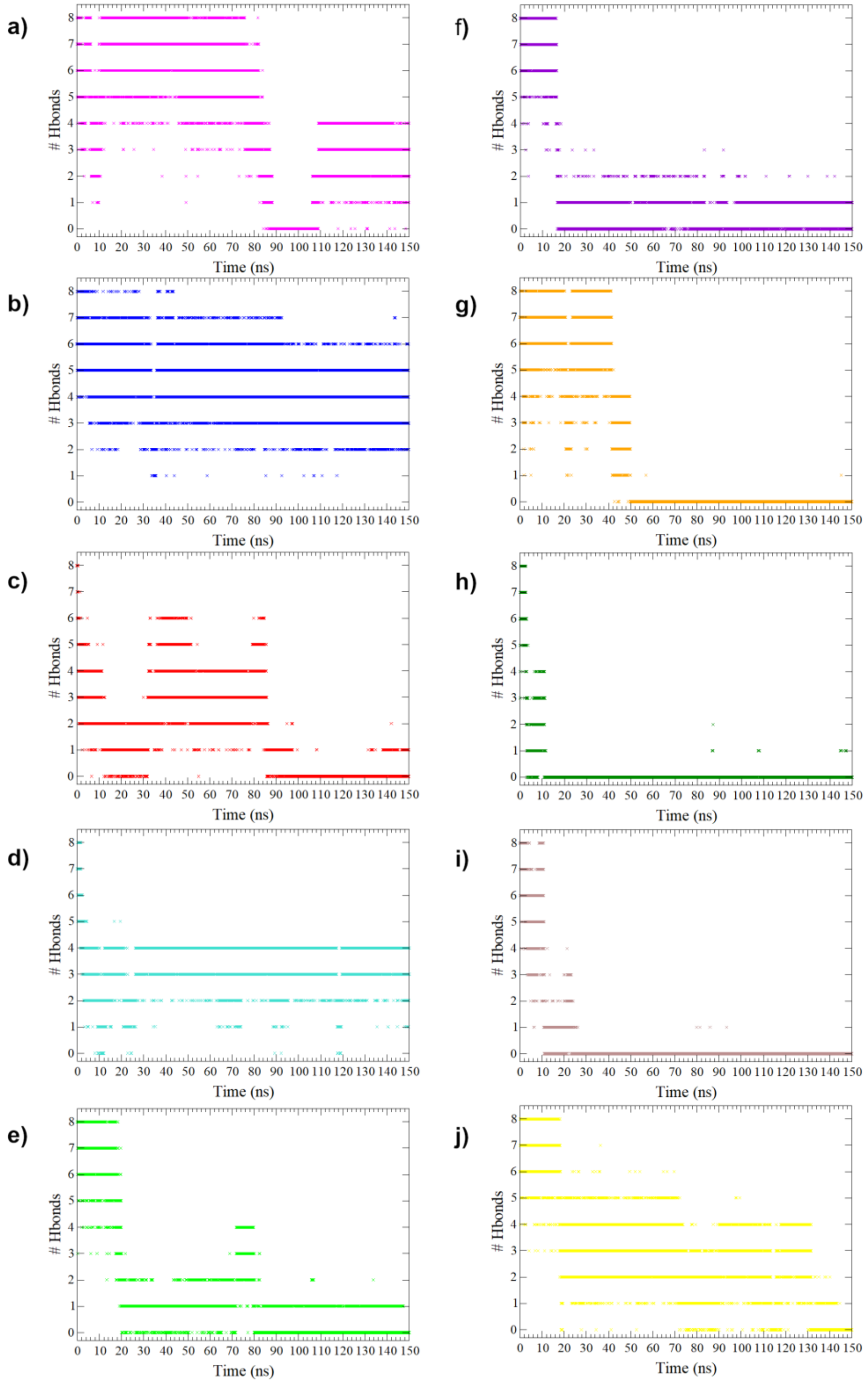

Figure S15. Time evolution of the total number of Hoogsteen H-bonds along the 13-mer trajectories. (A) 3-RG1_1; (B) 3-RG1_2; (C) 3-RG1_3; (D) 3-RG1_4; (E) 3-RG1_5; (F) 5-RG1_1; (G) 5-RG1_2; (H) 5-RG1_3; (I) 5-RG1_4; (J) 5-RG1_5.

Table S3. Fraction of Hoogsteen H-bonds along the 150 ns trajectories of the 13-mer models.

**Hbond**	**3-RG1 run 1**	**3-RG1 run 2**	**3-RG1 run 3**	**3-RG1 run 4**	**3-RG1 run 5**	**5-RG1 run 1**	**5-RG1 run 2**	**5-RG1 run 3**	**5-RG1 run 4**	**5-RG1 run 5**	**Hbond**
Triad1*						***Triad1***					
Strand a+b**						***Strand a+b***					
**G1-G5** N1...O6	0.64	0.5<br>0	0.23	0.90	0.50	0.11	0.22	0.02	0.05	0.13	**G3-G9** N1...O6
**G1-G5** N2...N7	0.78	0.9<br>6	0.42	0.95	0.30	0.12	0.26	0.02	0.05	0.12	**G3-G9** N2...N7
Strand b+c						***Strand b+c***					
**G5-G11** N1...O6	0.39	0.3<br>1	0.14	0.02	0.09	0.09	0.21	0.04	0.06	0.48	**G9-G12** N1...O6
**G5-G11** N2...N7	0.45	0.6<br>7	0.06	0.02	0.12	0.10	0.24	0.05	0.06	0.49	**G9-G12** N2...N7
Triad2						***Triad2***					
Strand a+b						***Strand a+b***					
**G2-G6** N1...O6	0.61	0.4<br>0	0.24	0.79	0.12	0.27	0.26	0.02	0.07	0.42	**G4-G10** N1...O6
**G2-G6** N2...N7	0.72	0.9<br>3	0.51	0.87	0.17	0.38	0.30	0.02	0.06	0.12	**G4-G10** N2...N7
Strand b+c						***Strand b+c***					
**G6-G12** N1...O6	0.33	0.5<br>4	0.09	0.01	0.09	0.08	0.19	0.04	0.06	0.72	**G10-G13** N1...O6
**G6-G12** N2...N7	0.48	0.2<br>1	0.00	0.01	0.10	0.10	0.24	0.03	0.05	0.48	**G10-G13** N2...N7

*Triad1 is the planar unit formed by three H-bonded guanosines including the 5'G, whereas triad2 contains the 3' G (see also Figure S6B,C).
**The strands, containing the Gs which form the two triad planes, are denoted as "a", "b" and "c" in a sequential manner by starting from the "a" strand which comprises the 5' G (Figure S6B,C).

A) B)

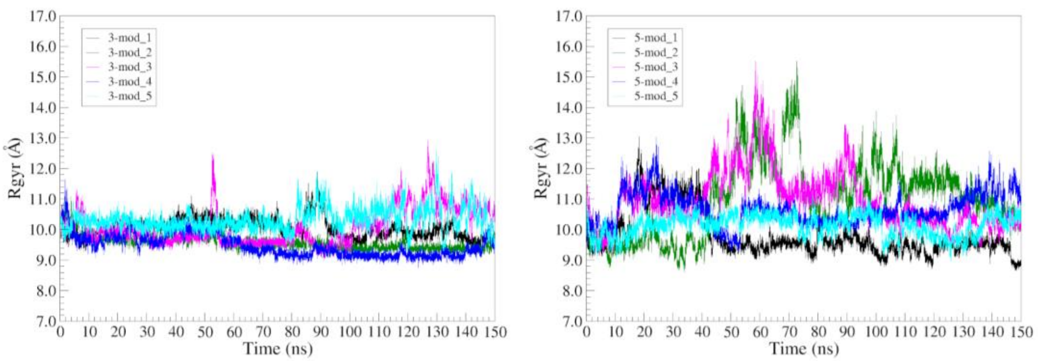

Figure S16. Time evolution of the radius of gyration (Rgyr). The Rgyr is calculated for all nucleotide atoms of the two-triad 13mer models for the five independent MD runs. (A) 3-RG1 simulations; (B) 5-RG1 simulations.

To better discriminate between the dominant conformations explored by the two different systems, 3-RG1 and 5-RG1, we have then joined the five independent trajectories into a single one (750 ns long) and applied a clustering procedure to find five clusters that group similar conformations based on all-atom RMSDs. We have then examined the representative structures for each of the clusters in order to find conformations similar to the starting G-triplex structure.

For the 3-RG1 system, the G-triplex structure is present as the first most abundant structure with a population of about 43% (Figure 4C of the main text). The triad planes are rather planar. The stacking of C7 below the G6 base is also retained from the starting structure as well as that of the U4 base which indeed improves its stacking above the G5 base. In the MD structure however, there is a new stacking interaction to the G stem involving the 3'end C13, which is now stacked below the G12 base. It is worth noting that in the ensemble of 3-RG1 structures the representative of the second highest populated cluster (32%) also retains some similarity with the G-triplex structure. Indeed, it shows the conserved parallel relative orientation of the three strands (a, b, and c) as well as the stacking of the two adjacent G bases along each strand, but with a large misalignment of the base planes.

On the other hand, for the 5-RG1 system, we cannot find a structure similar to the G-triplex architecture in the representative structures of the five clusters considered. Indeed, the dominant conformations are single stranded segments folded back onto themselves forming extensive base-stacking interactions and no G pairings by Hoogsteen H-bonds. The higher instability of the G-triplex structure in 5-RG1 is probably due to the presence of a single nucleotide (C11) which connects Gs in b and c strands and is in charge of the reverse of the chain orientation in order to have parallel b and c strands. Hence, 5-RG1 exhibits a tendency to align b and c strands in a continuous stretch of stacked bases including C11, thus destroying the triplex architecture.

References

[1] R. D. Gray, J. B. Chaires, *Curr. Protoc. Nucleic Acid Chem.* **2011**, *45*, DOI 10.1002/0471142700.nc1704s45.
[2] R. W. Hendler, R. I. Shrager, *J. Biochem. Biophys. Methods* **1994**, *28*, 1–33.
[3] Y. Zhao, D. G. Truhlar, *Acc. Chem. Res.* **2008**, *41*, 157–167.
[4] L. Martínez Fernández, F. Santoro, R. Improta, *Acc. Chem. Res.* **2022**, *55*, 2077–2087.
[5] R. Improta, F. Santoro, L. Blancafort, *Chem. Rev.* **2016**, *116*, 3540–3593.
[6] L. Martínez-Fernández, L. Esposito, R. Improta, *Photochem. Photobiol. Sci.* **2020**, *19*, 436–444.
[7] H. Asha, J. A. Green, L. Esposito, F. Santoro, R. Improta, *Chirality* **2023**, *35*, 298–310.
[8] H. Asha, J. A. Green, L. Martinez-Fernandez, L. Esposito, R. Improta, *Molecules* **2021**, *26*, 4789.
[9] J. A. Green, H. Asha, F. Santoro, R. Improta, *J. Chem. Theory Comput.* **2021**, *17*, 405–415.
[10] M. J. Frisch, G. W. Trucks, H. B. Schlegel, G. E. Scuseria, M. A. Robb, J. R. Cheeseman, G. Scalmani, V. Barone, G. A. Petersson, H. Nakatsuji, X. Li, M. Caricato, A. V. Marenich, J. Bloino, B. G. Janesko, R. Gomperts, B. Mennucci, H. P. Hratchian, J. V. Ortiz, A. F. Izmaylov, J. L. Sonnenberg, D. Williams-Young, F. Ding, F. Lipparini, F. Egidi, J. Goings, B. Peng, A. Petrone, T. Henderson, D. Ranasinghe, V. G. Zakrzewski, J. Gao, N. Rega, G. Zheng, W. Liang, M. Hada, M. Ehara, K. Toyota, R. Fukuda, J. Hasegawa, M. Ishida, T. Nakajima, Y. Honda, O. Kitao, H. Nakai, T. Vreven, K. Throssell, J. A. Montgomery, Jr., J. E. Peralta, F. Ogliaro, M. J. Bearpark, J. J. Heyd, E. N. Brothers, K. N. Kudin, V. N. Staroverov, T. A. Keith, R. Kobayashi, J. Normand, K. Raghavachari, A. P. Rendell, J. C. Burant, S. S. Iyengar, J. Tomasi, M. Cossi, J. M. Millam, M. Klene, C. Adamo, R. Cammi, J. W. Ochterski, R. L. Martin, K. Morokuma, O. Farkas, J. B. Foresman, and D. J. Fox, Gaussian 16 Revision C.01, **2016**, Gaussian Inc., Wallingford, CT, USA.
[11] M. Zgarbová, M. Otyepka, J. Šponer, A. Mládek, P. Banáš, T. E. I. Cheatham, P. Jurečka, *J. Chem. Theory Comput.* **2011**, *7*, 2886–2902.
[12] J. Tomasi, B. Mennucci, R. Cammi, *Chem. Rev.* **2005**, *105*, 2999–3094.
[13] M. Cossi, N. Rega, G. Scalmani, V. Barone, *J. Comput. Chem.* **2003**, *24*, 669–681.
[14] G. Scalmani, M. J. Frisch, *J. Chem. Phys.* **2010**, *132*, 114110.
[15] V. Karunakaran, K. Kleinermanns, R. Improta, S. A. Kovalenko, *J. Am. Chem. Soc.* **2009**, *131*, 5839–5850.
[16] F. J. Avila Ferrer, J. Cerezo, E. Stendardo, R. Improta, F. Santoro, *J. Chem. Theory Comput.* **2013**, *9*, 2072–2082.
[17] H. Asha, J. A. Green, L. Esposito, L. Martinez-Fernandez, F. Santoro, R. Improta, *J. Phys. Chem. B* **2022**, *126*, 10608–10621.
[18] D.A. Case, H.M. Aktulga, K. Belfon, I.Y. Ben-Shalom, J.T. Berryman, S.R. Brozell, D.S. Cerutti, T.E. Cheatham, III, G.A. Cisneros, V.W.D. Cruzeiro, T.A. Darden, R.E. Duke, G. Giambasu, M.K. Gilson, H. Gohlke, A.W. Goetz, R. Harris, S. Izadi, S.A. Izmailov, K. Kasavajhala, M.C. Kaymak, E. King, A. Kovalenko, T. Kurtzman, T.S. Lee, S. LeGrand, P. Li, C. Lin, J. Liu, T. Luchko, R. Luo, M. Machado, V. Man, M. Manathunga, K.M. Merz, Y. Miao, O. Mikhailovskii, G. Monard, H. Nguyen, K.A. O'Hearn, A. Onufriev, F. Pan, S. Pantano, R. Qi, A. Rahnamoun, D.R. Roe, A. Roitberg, C. Sagui, S. Schott-Verdugo, A. Shajan, J. Shen, C.L. Simmerling, N.R. Skrynnikov, J. Smith, J. Swails, R.C. Walker, J. Wang, J. Wang, H. Wei, R.M. Wolf, X. Wu, Y. Xiong, Y. Xue, D.M. York, S. Zhao, and P.A. Kollman, Amber, **2022**, University of California, San Francisco, CA, USA.
[19] J. Wang, P. Cieplak, P. A. Kollman, *J. Comput. Chem.* **2000**, *21*, 1049–1074.
[20] A. Pérez, I. Marchán, D. Svozil, J. Sponer, T. E. Cheatham, C. A. Laughton, M. Orozco, *Biophys. J.* **2007**, *92*, 3817–3829.
[21] W. L. Jorgensen, J. Chandrasekhar, J. D. Madura, R. W. Impey, M. L. Klein, *J. Chem. Phys.* **1983**, *79*, 926–935.
[22] I. S. Joung, T. E. I. Cheatham, *J. Phys. Chem. B* **2008**, *112*, 9020–9041.
[23] T. Miclot, C. Hognon, E. Bignon, A. Terenzi, M. Marazzi, G. Barone, A. Monari, *J. Phys. Chem. Lett.* **2021**, *12*, 10277–10283.
[24] J. Åqvist, P. Wennerström, M. Nervall, S. Bjelic, B. O. Brandsdal, *Chem. Phys. Lett.* **2004**, *384*, 288–294.
[25] T. Darden, D. York, L. Pedersen, *J. Chem. Phys.* **1993**, *98*, 10089–10092.
[26] J.-P. Ryckaert, G. Ciccotti, H. J. C. Berendsen, *J. Comput. Phys.* **1977**, *23*, 327–341.
[27] D. R. Roe, T. E. I. Cheatham, *J. Chem. Theory Comput.* **2013**, *9*, 3084–3095.
[28] W. Humphrey, A. Dalke, K. Schulten, *J. Mol. Graph.* **1996**, *14*, 33–38.
[29] Schrödinger, LLC. The PyMOL Molecular Graphics System, Version 1.8.2.0, **2015**, New York, NY, USA.
[30] R. Improta, *Chem. – Eur. J.* **2014**, *20*, 8106–8115.
